# Genotype‐aggregated planting improves yield in Jerusalem artichoke (*Helianthus tuberosus*) due to self/non‐self‐discrimination

**DOI:** 10.1111/eva.12735

**Published:** 2018-11-29

**Authors:** Yuya Fukano, Wei Guo, Koji Noshita, Shoko Hashida, Shotaka Kamikawa

**Affiliations:** ^1^ Graduate School of Agricultural and Life Sciences The University of Tokyo Nishitokyo Japan; ^2^ Japan Science and Technology Agency (JST) Precursory Research for Embryonic Science and Technology (PRESTO) Saitama Japan

**Keywords:** agriculture, kin recognition, plant communication, tuber

## Abstract

Accumulating evidence indicates that plants are capable of self/non‐self and kin/stranger discrimination. Plants increase biomass of and resource allocation to roots when they encounter roots of conspecific non‐self‐neighbors, but not when they encounter self roots. Root proliferation usually occurs at the expense of reproductive investment. Therefore, if clonal crops are capable of self/non‐self‐discrimination, spatially aggregated planting with seedlings of the same genotype may decrease root proliferation and produce a higher yield than planting without considering seedling genotype. To test this idea, we grew *Helianthus tuberosus* (Jerusalem artichoke) in pot and field conditions and examined self/non‐self‐discrimination and the effectiveness of genotype‐aggregated planting. Plants grown in self pairs allocated less to root biomass than plants grown in non‐self pairs in both pot and field conditions; in field conditions, the self pairs produced 40% more tubers by weight than the non‐self pairs. When six sprouts from seed tuber of two different genotypes were grown together, with the two genotypes planted aggregately (AGG) or alternately (ALT), plants in the AGG group produced 14% more tubers than plants in the ALT group. These results suggest that spatial aggregation of genotypes increases tuber production in *H. tuberosus*. Because we found no evidence for trade‐offs between root biomass and tuber production, suppression of root proliferation may not be the only mechanism behind the benefits of genotype aggregation. By applying the concept of self/non‐self‐discrimination, farmers can increase crop production without additional external inputs or expansion of agricultural land use.

## 1 INTRODUCTION

In agricultural systems, crop growth and yield are reduced by interspecific competition (between crops and weeds) and intraspecific competition (among plants of the same crop; Cousens, 1985; Deng et al., 2012; Kropff & Spitters, 1991). Interspecific competition can be effectively reduced by conventional weed management practices, such as herbicide application and soil tillage (Chhokar, Sharma, Jat, Pundir, & Gathala, 2007; Wrucke & Arnold, 1985). However, intraspecific competition is more difficult to reduce in agricultural ecosystems. Therefore, breeding and cultivation methods that mitigate intraspecific competition to increase crop yield have long been studied (Deng et al., 2012; Donald, 1963, 1968; Sedgley, 1991).

Accumulating evidence suggests that the degree of intraspecific competition between plants depends on the identity of the neighboring plants (Chen, During, & Anten, 2012; Karban, 2015; Novoplansky, 2009). Several species have a plastic response, allowing them to allocate additional resources to root biomass when they face belowground intraspecific competition (Gersani, Brown, O'Brien, Maina, & Abramsky, ; Maina, Brown, & Gersani, 2002; O'Brien, Gersani, & Brown, 2005; Smyčka & Herben, 2017 but see Hess & De Kroon, 2007). Plants that use the “tragedy of the commons” rooting strategy (TOC strategy) maximize the speed of nutrient uptake under competitive conditions. However, this proliferation of roots in shared space results in a lower reproductive yield than if both plants restrained their root production to match resource availability (Gersani et al., ; Maina et al., 2002; O'Brien et al., 2005). For example, O'Brien et al. (2005) showed that pairs of *Pisum sativum* plants in pots of volume V increase root biomass and decrease reproductive organ biomass (pod mass) compared to single plants of *P. sativum* in pots of volume V/2.

The TOC strategy is related to self/non‐self and kin discrimination by roots (Falik, Reides, Gersani, & Novoplansky, 2003; Holzapfel & Alpert, 2003; Novoplansky, 2009). Several papers have reported that plants increase root biomass in the presence of non‐self and non‐kin neighboring plants, but not in the presence of self and kin roots (Chen et al., 2012; Depuydt, 2014; Semchenko, Saar, & Lepik, 2014; Yamawo, Sato, & Mukai, 2017). For example, *Buchloe dactyloides* (Nutt.) Engelm plants can discriminate between self and non‐self roots and develop more and longer roots in the presence of non‐self‐neighbors (Gruntman & Novoplansky, 2004). Dudley and File (2007) found that *Cakile edentula* (Bigelow) Hook in non‐kin groups allocated more to their fine root mass than did plants in kin groups when they competed for belowground resources. Thus, plants increase root proliferation in the presence of genetically distant or unrelated neighboring competitors compared to those grown with genetically related neighbors.

Regulation of root proliferation can affect crop yield, which is of interest in crop science (Depuydt, 2014). Firstly, root proliferation decreases resource allocation to reproductive organs because of a trade‐off (Gersani et al., ; Maina et al., 2002). This change in resource allocation will reduce the yield of reproductive organs such as tubers, bulbs, fruits, and seeds at the individual level. Secondly, root proliferation can increase belowground competition among neighboring plants. The increase in belowground competition may lead to a decrease in total biomass production at the population level, decreasing the yield. Thus, suppression of root proliferation within a crop may effectively increase yield. If root proliferation is affected by genotype‐based self/non‐self‐discrimination of neighboring plants, the spatial structure of crop genotypes may change the degree of root proliferation, belowground competition, and yield. Spatially aggregated planting of seedlings of the same genotype, compared with planting without considering seedling genotype, may mitigate wasteful competition among seedlings and may increase yield. On the other hand, if root proliferation increases the acquisition of water or nutrients without increasing competition with neighbors, root proliferation might increase tuber production. In this case, spatially aggregated planting of the same genotype would result in lower root production and yield than planting without considering genotype.

Root proliferation is not the only response to non‐self plants. Plants can show various responses in root growth pattern depending on neighbor identity. For example, Holzapfel and Alpert (2003) reported that connected clonal plants of the wild strawberry (*Fragaria chiloensis* L.) segregate their roots. Root segregation of self plants can increase resource capture efficiency, plant performance, and fitness. Plants discriminate between kin and non‐kin neighbors and induce plastic responses also in aboveground vegetative parts. Crepy and Casal (2015) reported that *Arabidopsis thaliana* L. plants growing close to a kin neighbor change the orientation of leaf growth and reduce kin competition for light, which may help pairs of kin individuals to achieve higher growth rate and reproduction than in non‐kin pairs. Thus, regardless of the type of the responses to neighbors (root proliferation, root segregation, or leaf orientation), spatially aggregated planting of seedlings of the same genotype may mitigate wasteful competition among seedlings and increase yield.

It should be noted that the genetic diversity of many crops has been reduced by the process of cultivation, resulting in populations that are uniformly highly related (Murphy, Acker, Rajcan, & Swanton, 2017). Under such circumstances, self/non‐self‐discrimination is rarely advantageous, and thus, it may be lost during crop domestication. Many modern varieties have lost most of their genetic diversity within populations and are genetically homogenous compared to their wild relatives (Lam et al., 2010). A certain degree of genetic diversity remains in open‐pollinated crops, heirloom varieties, landraces, and crops that have only been weakly domesticated (Reif et al., 2005; RodrÍGuez‐Burruezo, Prohens, RosellÓ, & Nuez, 2005). For cultivation of such “primitive” crops, genotype‐aggregated planting of seedlings may be effective for increasing yield.

In this study, we tested the effectiveness of genotype‐aggregated planting of *Helianthus tuberosus *L. (Jerusalem artichoke). *Helianthus tuberosus *is native to North America and is thought to have originated in the Great Lakes area (Swanton, Cavers, Clementsl, & Moore, 1992). Because it produces large quantities of edible tubers, *H. tuberosus* was an important crop for native North Americans prior to European contact (Kays & Nottingham, 2007). After being introduced to European countries in the early sixteenth century, *H. tuberosus* spread throughout the Mediterranean region and became popular as a root crop. After potato (*Solanum tuberosum*) cultivation became common in the mid‐eighteenth century, the relative importance of *H. tuberosus* decreased (Kays & Nottingham, 2007). Recently, however, cultivation of *H. tuberosus* has been regaining popularity owing to its potential health benefits; the tubers are rich in the carbohydrate inulin, which is a water‐soluble dietary fiber, as opposed to starch which is not water‐soluble and has a higher glycemic index (Kleessen et al., 2007). In addition, *H. tuberosus* is being studied as a candidate crop for use in biofuels and livestock feed because of its high production of above‐ and belowground biomass (Cheng et al., 2009).

*Helianthus tuberosus* can reproduce either sexually through seeds or clonally via tubers. Seedlings or sprouts germinate from seeds and tubers in the spring and take approximately 130 days to produce mature seeds and tubers (Swanton et al., 1992). The flowers are pollinated by bees and are outcrossing (Swanton et al., 1992). Several studies have reported a high level of genetic diversity among clones and populations in physiological, morphological, and life history traits (Kays & Kultur, 2005; Puttha et al., 2012; Swanton et al., 1992). Natural and artificial selection may favor self/non‐self‐discrimination to reduce wasteful competition with self‐neighbors.

We investigated the effects of self/non‐self‐discrimination on biomass allocation and the degree of intraspecific competition in *H. tuberosus* by comparing growth and biomass between plants of the same genotype (self pairs) and those of different genotypes (non‐self pairs). We also investigated the effect of genotype‐aggregated planting on yield in *H. tuberosus*. To quantify the degree of competition between pairs, we measured inequalities in size and biomass. Inequalities in size and biomass among competing individuals are thought to be caused by asymmetric competition that is tightly related to intraspecific competition (Biernaskie, 2011; Weiner & Thomas, 1986). To examine the effectiveness of genotype‐aggregated transplanting, we compared six sprouts grown from tubers of two genotypes, planted either with genotypes aggregated (AGG) or alternated (ALT). Specifically, we addressed the following questions. (a) Do plants of non‐self pairs show increased root biomass and allocation compared with plants of self pairs? (b) Are biomass and size inequality greater between plants in non‐self pairs than between plants in self pairs? (c) Is tuber production higher in self pairs and in AGG plantings than in non‐self pairs and ALT plantings? Finally, we focused on the trade‐offs between tuber production and other traits. If the benefit of genotype aggregation comes from the suppression of root proliferation in response to a non‐self‐neighbor, plants in non‐self pairs and ALT planting will increase root biomass and allocation and show a negative correlation between root and tuber biomasses. If the benefit of genotype aggregation comes from increased resource acquisition in the presence of a self‐neighbor (such as self‐plant root segregation; Holzapfel & Alpert, 2003), plants will not increase root biomass and allocation and will show a positive correlation between root and tuber biomasses. If the benefit of genotype aggregation comes from self‐discrimination in aboveground vegetative parts, plants will change aboveground biomass and allocation depending on the identity of neighbor and show a positive correlation between aboveground and tuber biomasses.

## 2 MATERIALS AND METHODS

To evaluate the ability for self/non‐self‐discrimination and the effectiveness of AGG transplanting on tuber production in *H. tuberosus*, we performed three types of experiments. In the pot experiment, temporal changes in biomass allocation according to neighboring plant type under controlled soil conditions were investigated. In the field experiment with single and paired plants, phenotypic plasticity in biomass production and growth inequality was investigated according to neighboring plant type. In the field experiment with group planting, the effect of AGG planting on tuber production was tested.

### 2.1 Pot experiment with paired plants

This experiment was conducted at the Fuchu campus of Tokyo University of Agriculture and Technology (TUAT) in 2015 and at the Institute for Sustainable Agro‐Ecosystem Services (ISAS) of Tokyo University in 2017. We purchased seed tubers from three private farms in Tochigi, Chiba, and Gunma Prefectures, Japan. These farms are at least 80 km apart from each other and individual plants from each farm differed in various morphological traits of shoots and tubers (our unpublished observation). Thus, the plants from each farm were treated as a distinct population. We treated an individual seed tuber as a genetically distinct entity (genotype). Each tuber was divided into two to six fragments depending on size. All divided tubers were weighed (12.0–38.0 g) and planted into individual nursery pots (6 cm in diameter; 0.3 L volume) with commercial soil mixture “Golden” (Iris Ohyama Co., Miyagi, Japan). In total, we obtained 497 sprouts of 192 genotypes in 2015 and 567 sprouts of 174 genotypes in 2017.

We randomly selected and transplanted them into the experimental pots (18 cm in diameter; 3.5 L volume), using three types of pairings (Supporting Information Figure S1): pairs of the same genotype (SG), pairs of different genotypes from the same population (DG), and pairs of different genotypes from different populations (DP). We treated SG pairs as self pairs and DG and DP pairs as non‐self pairs (Supporting Information Figure S1a). The commercial soil mixture “Golden” was used in experimental pots. The distance between paired sprouts was 10 cm. On 28 April 2015, we planted 120 plants in 20 pairs each of SG, DG, and DP outdoors. On 5 May 2017, we planted 118 plants in 19 SG pairs, 20 DG pairs, and 20 DP pairs in a greenhouse.

We measured the fresh weight of the aboveground parts, roots, newly formed tubers, and seed tuber portions of the plants, as well as plant height. At 40 days after transplantation (DAT) in 2015 and 30 DAT 2017, we randomly selected 10 pairs of each type for destructive measurement. The remaining plants were measured at 80 DAT in 2015 and 60 DAT in 2017. After drying the plants at 80°C for 7 days, we measured the dry weight.

### 2.2 Field experiment with single and paired plants

We selected 60 sprouts of 50 genotypes from the nursery pots and planted them in 10 pairs each of SG, DG, and DP. These pairs were transplanted randomly into three‐row plots of a crop field at ISAS on 28 April 2017. The plots were covered with plastic mulch film (60 cm width). The distance between sprouts within each pair was 20 cm (not shown), and the distance between pairs was 1 m (Supporting Information Figure S1b). In addition, we selected 60 sprouts of the same genotypes as the transplanted paired sprouts and transplanted them individually on both sides of the plot that contained the paired plants (Supporting Information Figure S1b). This experimental design allowed us to evaluate the phenotypic plasticity of each genotype in response to the neighboring plants (SG, DG, and DP) by comparing plants grown individually and those of the same genotypes grown in pairs. Plant height and stem diameter were measured every 30 DAT, until August 28.

We harvested the aboveground portions of the plants at the end of October and belowground portions (roots and tubers) at the end of November; we used hand hoes to dig to a depth of 30 cm in 1.5 m × 1.5 m quadrats centered on the plants. The fresh weight of the aboveground portions was measured immediately after harvest. The fresh weight of roots and tubers was measured after removing surface mud and drying in the shade for 1 day. For paired plants, it was impossible to determine which parent plant the collected tubers were derived from. Thus, the fresh weight of the tubers of each plant was determined by halving the total tuber weight for the plant pair.

### 2.3 Field experiment with group planting

We selected 246 sprouts of 82 genotypes from the nursery pots and assigned them to 22 AGG and 19 ALT groups of six sprouts (two genotypes × three sprouts). In each AGG group, all three sprouts of the same genotype were transplanted aggregately (Supporting Information Figure S1c). In each ALT group, sprouts of different genotypes were transplanted alternately (Supporting Information Figure S1c). We randomly assigned the seedling groups to 10‐row plots at the ISAS field on 2 May 2017. The six sprouts in each group were transplanted in a single line. The distance between sprouts within each group was 10 cm, and the distance between groups was 250 cm, measured from center to center (Supporting Information Figure S1c).

At the end of October, we harvested the aboveground portions of each group. Belowground portions were harvested as described above except that 2.5 m × 2 m quadrats around each group were dug using a backhoe and hand hoes. We measured the fresh weight of the aboveground and belowground portions of each group as above.

### 2.4 Statistical analysis

For analysis of the pot experiment, to determine whether neighbor type affected growth and tuber production, we used generalized linear mixed models (GLMMs) with a Gaussian distribution. The final plant height, dry weight of roots, aboveground parts, and newly formed tubers, and total dry weight were treated as response variables. The self‐identity (self or non‐self), type of non‐self (DG or DP) nested in non‐self, pot ID, and initial tuber weight were treated as explanatory variables, and source population was treated as a random effect. To determine whether neighbor type affected resource allocation to roots and tubers, we incorporated correlated traits as covariates (van Noordwijk & de Jong, 1986); dry weight values of roots, newly formed tubers, and belowground parts were treated as response variables. For the analysis of allocation to roots, we used the explanatory variables mentioned above and also aboveground and total weight as explanatory variables. For the analysis of allocation to tubers, root and aboveground weight were added as explanatory variables. For the analysis of allocation to belowground parts, total weight was added as an explanatory variable. We used the lmer function of the software package R (Bates, Maechler, Bolker, & Walker, 2014; R Development Core Team, 2010). Likelihood ratio tests were used to evaluate the significance of the explanatory variables.

For the field experiment with single and paired plants, we conducted two analyses to examine whether neighbor type affected the growth and resource allocation. First, we investigated how growth and tuber production changed according to neighbor type. In this analysis, the final plant height, the final stem diameter, fresh weights of roots, aboveground parts, and newly formed tubers, and total fresh weight were treated as response variables. The data for both the single and paired plants were fitted to the GLMMs with a Gaussian distribution. The growth condition (single or paired), self‐identity (self or non‐self), type of non‐self (DG or DP) nested in non‐self, genotype, initial tuber weight, and the interaction between growth condition × self‐identity and growth condition × type of non‐self were treated as explanatory variables. For the analysis of allocation to roots, tuber, and belowground weight, we added the same variables to the explanatory variables as in the pot experiment. Source population was treated as a random effect. The interaction terms were used to determine whether *H. tuberosus* exhibited different reaction norms under competition with different types of paired neighbors. Second, we used the data for the paired plants to examine whether neighbor type affected the growth and resource allocation, as in the pot experiment. The models were fitted using self‐identity (self or non‐self), type of non‐self (DG or DP) nested in non‐self, pair ID, and initial tuber weight as explanatory variables; source population was treated as a random effect.

To examine whether neighbor type caused size and biomass inequalities between paired plants, we calculated the coefficients of variation (CVs) of the height, stem diameter, aboveground fresh weight, and root fresh weight between paired plants. We excluded tuber weight from this analysis because it was impossible to determine which plant produced which tubers. If intraspecific competition was higher in non‐self pairs than self pairs, it was expected that the CVs in the non‐self pairs (DG and DP) would be higher than in the self pairs (SG). However, a simple comparison between CVs in the self and the non‐self pairs is not appropriate to quantify the degree of competition because the genetic variation of non‐self pairs should be higher than that of self pairs. In this case, it was impossible to determine whether differences in CVs between self and non‐self pairs were due to genetic differences or to differences in competition. Therefore, we compared the CVs of two plants grown in paired conditions and the CVs of two plants of the same genotype grown individually. If competition between non‐self pairs is higher than that between self pairs, it is expected that the difference between the CVs of two plants grown as a pair (with competition) and the CVs of two plants of these same genotypes grown alone (without competition) would be greater in non‐self pairs than in self pairs. For this analysis, the CVs for each measurement were fitted to the generalized linear models (GLMs) using a Gaussian distribution. The growth condition (single or paired), self‐identity (self or non‐self), type of non‐self (DG or DP) nested in non‐self, genotype, and the interaction between growth condition × self‐identity and growth condition × type of non‐self were treated as explanatory variables.

In the analysis of the field experiment with group planting, we used GLMMs with a Gaussian distribution. The fresh weight of the aboveground parts, roots, and tubers and total fresh weight were treated as response variables. The type of group (AGG or ALT) and the plot ID were treated as an explanatory variable and a random effect, respectively. For the analysis of allocation to roots, tubers, and belowground parts, we used the same explanatory variables as in the pot experiment.

## 3 RESULTS

### 3.1 Pot experiment

**Figure 1:**
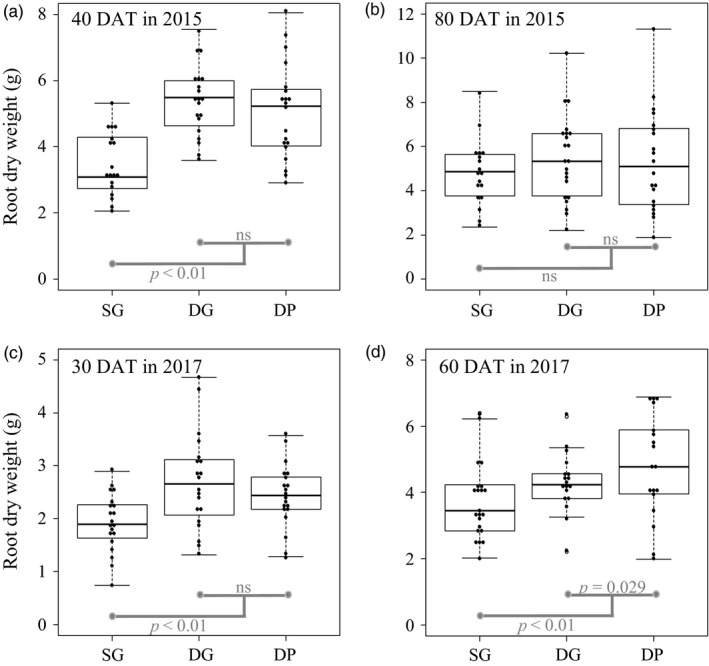
Beeswarm and box plots of root dry weight in the pot experiments. Plants were grown with plants of the same genotype (SG), different genotype (DG), and different population (DP) for 40 days after transplantation (DAT), 80 DAT in 2015 (a and b, respectively), 30 DAT and 60 DAT in 2017 (c and d, respectively)

The summary of the results is shown in Table 1. The root dry weight of plants in self pairs was lower than that of non‐self pairs at 40 DAT in 2015 and 30 and 60 DAT in 2017 (Figure 1, Table 1, Supporting Information Table S1). Plants in self pairs were taller than those in non‐self pairs at 60 DAT in 2017 (Table 1, Supporting Information Table S1, Figure S2). The DG and DP pairs differed significantly in height at 80 DAT in 2015, in root weight at 60 DAT in 2017 and in tuber weight at 30 DAT in 2017 (Table 1 and Supporting Information Table S1). There were no significant differences between self and non‐self pairs and DG and DP pairs in other comparisons (Table 1, Supporting Information Table S1, Figures [Link], [Link], [Link]–S5).

**Table 1:** Generalized linear mixed model analysis of the pot experiment using paired plants at 40 and 80 days after transplantation (DAT) in 2015 and 30 and 60 DAT in 2017

	2015				2017			
	40 DAT		80 DAT		30 DAT		60 DAT	
Explanatory variables	Self‐identity	Type of non‐self	Self‐identity	Type of non‐self	Self‐identity	Type of non‐self	Self‐identity	Type of non‐self
Height	Ns	Ns	Ns	DG < DP**	Ns	Ns	Self > non‐self**	Ns
Aboveground dry weight	Ns	DG < DP*	Ns	Ns	Ns	Ns	Ns	Ns
Root dry weight	Self < non‐self***	Ns	Ns	Ns	Self < non‐self***	Ns	Self < non‐self***	DG <DP **
Tuber dry weight	Ns	Ns	Ns	Ns	Ns	DG < DP**	Ns	Ns
Total dry weight	Ns	Ns	Ns	Ns	Self < non‐self*	Ns	Ns	Ns
Allocation to tuber	Self > non‐self*	Ns	Ns	Ns	Self > non‐self**	Ns	Ns	Ns
Allocation to root	Self < non‐self***	Ns	Self < non‐self***	Ns	Self < non‐self***	Ns	Self < non‐self***	Ns
Allocation to belowground	Self < non‐self***	Ns	Ns	Ns	Self < non‐self***	Ns	Ns	Ns

Note

The relationship between plants grown with different types of neighboring plants and the significance of the differences in each trait are shown. DG and DP represent plants grown with plants of a different genotype from the same population and those from different populations, respectively. Asterisks indicate significant differences between types (^*^ *p* < 0.1, ^**^ *p* < 0.05, ^***^ *p* < 0.01).

The allocation to tubers was higher in self pairs than in non‐self pairs at 30 DAT in 2017 (Table 1, Supporting Information Table S1, Figure S6). The allocation to roots was lower in self pairs than in non‐self pairs at 40 and 80 DAT in 2015 and 30 and 60 DAT in 2017 (Table 1, Supporting Information Table S1, Figure S7). The allocation to belowground parts was lower in self pairs than in non‐self pairs at 40 DAT in 2015 and 30 DAT in 2017 (Table 1, Supporting Information Table S1, Figure S8). In all cases, allocation to tubers was not affected by root fresh weight (Supporting Information Table S1).

### 3.2 Field experiment with single and paired plants

**Figure 2:**
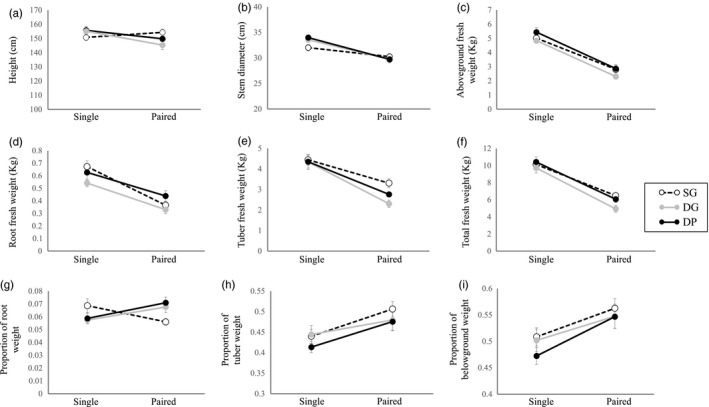
Plant height (a), stem diameter (b), aboveground fresh weight (c), root fresh weight (d) tuber fresh weight (e), total fresh weight (f), root weight divided by total weight (g), tuber weight divided by total weight (h), and belowground weight divided by total weight (i) of plants grown in single and paired conditions (mean ± SE) in the field experiment. Black dashed, gray, and black solid lines represent phenotypic differences between single plants (left) and plants grown with plants of the same genotype (SG), different genotype (DG), or different population (DP) in pairs (right), respectively

The summary of the results is shown in Table 2. Significant interactions between self‐identity (self or non‐self) and growth conditions (single or paired) were detected in height, stem diameter, tuber weight, allocation to tubers, and allocation to roots (Figure 2, Table 2, Supporting Information Table S2). The interaction between self‐identity and root fresh weight was marginally significant (*p* = 0.082, Table 2, Supporting Information Table S2). The plant height and tuber fresh weight of self pairs were higher than those of non‐self pairs (Figure 2a and e, Table 2, Supporting Information Table S3). Plants in self pairs produced 40% more tubers by fresh weight than plants in non‐self pairs (Figure 2e). No differences were detected between self pairs and non‐self pairs in stem diameter, aboveground fresh weight, or root fresh weight (Figure 2b‐d, Table 2, Supporting Information Table S3). Self pairs showed higher allocation to tubers and lower allocation to roots and belowground parts than non‐self pairs (Figure 2g–i, Supporting Information Figure S9a, Table 2, Supporting Information Table S3). Tuber fresh weight was positively correlated with root fresh weight but did not correlate with aboveground fresh weight (deviance = 11.661, *p* = 0.001 for root fresh weight, deviance = 1.034, *p* = 0.309 for aboveground fresh weight, Supporting Information Table S3).

**Table 2:** Generalized linear mixed model analysis of the field experiment using single and paired plants

Explanatory variables	Interactions terms	Comparisons among types in the paired plants	
		Self‐identity	DG versus DP
Height	***	Self > non‐self**	Ns
Stem diameter	**	Ns	Ns
Aboveground fresh weight	Ns	Ns	Ns
Root fresh weight	*	Ns	DG < DP**
Tuber fresh weight	**	Self > non‐self**	Ns
Total fresh weight	Ns	Self > non‐self*	DG < DP*
Allocation to tuber	***	Self > non‐self***	Ns
Allocation to root	***	Self < non‐self***	Ns
Allocation to belowground	Ns	Self < non‐self***	Ns
CV of height	Ns	Ns	Ns
CV of stem	Ns	Ns	Ns
CV of root fresh weight	*	Self < non‐self***	Ns
CV of aboveground fresh weight	**	Self < non‐self***	Ns

Note

The second column shows the significance of the interaction terms between self‐identity (self or non‐self) and growing condition (single and paired). The right two columns show the relationships between plants grown with different types of neighboring plants and whether significant differences were observed in each trait. DG and DP represent plants grown with plants of a different genotype from the same population and those from a different population, respectively. Asterisks indicate significant effects of interaction terms and differences between types (^*^ *p* < 0.1, ^**^ *p* < 0.05, ^***^ *p* < 0.01).

**Figure 3:**
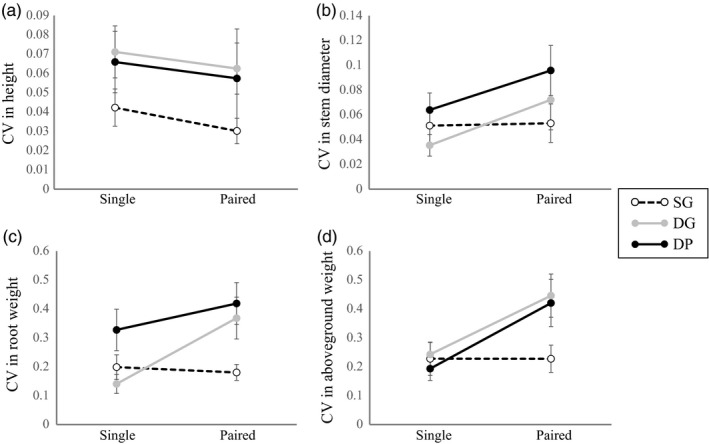
Coefficients of variance (CV) of height (a), stem diameter (b), root fresh weight (c), and aboveground fresh weight (d) between two plants grown under single and paired conditions (mean ± SE) in the field experiment. Black dashed, gray, and black solid lines represent differences between single plants (left) and plants grown with plants of the same genotype (SG), different genotype (DG), or different population (DP) in pairs (right), respectively

Significant interactions between self‐identity (self or non‐self) and growth conditions (single or paired) were detected in CVs of aboveground weight (Figure 3d, Table 2, Supporting Information Table S2). The interaction between self‐identity and growth conditions on root fresh weight was marginally significant (*p* = 0.083, Figure 3c, Table 2, Supporting Information Table S2). The CVs of the aboveground portions and root weight in self pairs were smaller than those of non‐self pairs (Figure 3c and d, Table 2, Supporting Information Table S3).

### 3.3 Field experiment with group planting

**Figure 4:**
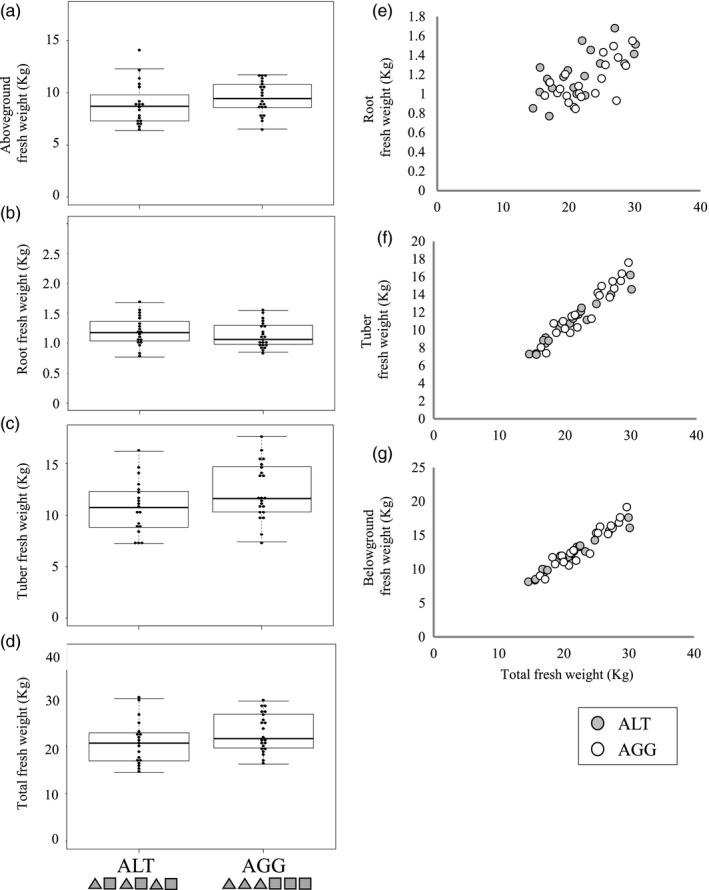
Beeswarm and box plots of aboveground fresh weight (a), root fresh weight (b), tuber fresh weight (c), total fresh weight (d), the relationship between total fresh weight and root fresh weight (e), tuber fresh weight (f), and belowground fresh weight (g) in six plants from the alternate planting (ALT) and aggregate planting (AGG) groups. In the AGG group, all three sprouts of the same genotype were transplanted aggregately. In the ALT group, sprouts of different genotypes were transplanted alternately

The fresh weight of tubers was greater in the AGG group than in the ALT group (Figure 4c, Supporting Information Table S4). Plants in the AGG group produced 14% more tubers than plants in the ALT group. The AGG group showed smaller allocation to roots (Figure 4e, Supporting Information Table S4) and greater allocation to tubers (Figure 4f, Supporting Information Figure S9b, Table S4) than the ALT group. No differences were detected between the AGG and ALT groups in aboveground fresh weight, root fresh weight, total fresh weight, or allocation to belowground parts (Supporting Information Table S4, Figure 4a, b, d, and g). Tuber weight was positively correlated with both the aboveground and root weight (deviance = 7.750, *p* = 0.005 for root fresh weight, deviance = 14.601, *p* < 0.001 for aboveground fresh weight, Supporting Information Table S4).

## 4 DISCUSSION

We examined the effect of self/non‐self‐discrimination on biomass allocation and investigated whether genotype‐aggregated transplanting could increase the yield of *H. tuberosus*. We found that plants in self pairs allocated less biomass to roots than plants in non‐self pairs in both pot and field conditions (Figures 1 and 2). For paired plants, the CVs of aboveground portions and roots were higher in non‐self pairs than in self pairs (Figure 3); for plants grown singly, the CVs did not differ significantly between self and non‐self pairs (Supporting Information Table S5). Thus, non‐self pairs experienced a higher level of intraspecific competition than self pairs. Finally, genotype‐aggregated planting increased the yield of *H. tuberosus*; plants in self pairs produced 40% more tubers than plants in non‐self pairs (Figure 2e), and plants in the AGG group produced 14% more tubers than plants in the ALT group (Figure 4c).

Although these results demonstrate the benefits of genotype aggregation for tuber production, the biological mechanisms behind these phenomena seem to be complicated. If the increase in tuber production in self pairs and the AGG group is caused by the suppression of root proliferation of the neighboring plant, plants in non‐self pairs and ALT planting will increase root biomass and allocation and there should be a negative correlation between root and tuber biomasses. However, while the non‐self pairs and ALT group showed increased root biomass and allocation, the correlation between root and tuber biomass was not significant in the pot experiment (Supporting Information Table S1) and was positive in the field experiment (Supporting Information Table S3). These results suggest that suppression of root proliferation is not the only mechanism of increased tuber production in genotype‐aggregated planting and but also other mechanisms exist, such as increased efficiency of resource acquisition. Plants of *H. tuberosus* might induce root segregation of self plants, as reported in wild strawberry *F. chiloensis *(Holzapfel & Alpert, 2003), which might increase the resource acquisition efficiency. Responses in the aboveground part may also be accounted for the benefit of genotype aggregation. In the field experiment with single and paired plants, plants in self pairs were taller than those in non‐self pairs and CV of aboveground biomass in self pairs was less than in non‐self pairs. These results imply that aboveground competition is lower in self pairs than in non‐self pairs. Because the growth of belowground parts directly depends on light acquisition by the aboveground parts, lower light competition in self pairs might facilitate tuber production. Actually, tuber fresh weight in the field experiment with group planting was positively correlated with the aboveground weight (Supporting Information Table S4). Further studies that would examine the response of the spatial distribution and allocation of roots and vegetative parts are needed to identify the causal mechanisms of the benefit of genotype aggregation.

Plants’ ability to discriminate between self and non‐self‐neighbors has been studied in various taxa, including several crop species (Chen et al., 2012; Depuydt, 2014; Karban, 2015; Murphy et al., 2017). Most of these studies used experiments in which the volume of belowground resources was limited, such as pot cultivation (but see Fang, Gao, Deng, Chen, & Liao, 2011). In our experiments, measurements in plants grown in pots suggested that self/non‐self‐discrimination was less likely to be detected under these conditions as the number of DAT increased in both years (Figure 1, Supporting Information Figure [Link], [Link], [Link], [Link], [Link], [Link], [Link]–S8 & Table 1). Because root mass of paired plants reached saturation in the pots, intermixing in the later DAT, self/non‐self‐discrimination may have been difficult to detect. To determine the importance of self/non‐self‐discrimination in agricultural and ecological systems, further field studies need to be conducted throughout the growing season.

The physiological mechanisms involved in self and kin discrimination among plants are largely unknown (Chen et al., 2012; Depuydt, 2014), although chemical substances in the root exudate may be involved (Biedrzycki, Jilany, Dudley, & Bais, 2010; Semchenko et al., 2014; Yamawo et al., 2017). These substances can be divided into two types: those indicating genetic differences and those indicating epigenetic differences. Evidence that plants can discriminate between clones, relatives, and cultivars suggests that chemicals derived from genetic differences enable discrimination (Dudley & File, 2007; Fang et al., 2013; Karban & Shiojiri, 2009; Murphy et al., 2017; Yamawo et al., 2017). On the other hand, many studies of self/non‐self‐discrimination reported the importance of physiological coordination; plants can discriminate severed and connected plants of the same genotype (Chen et al., 2012; Depuydt, 2014; Falik, de Kroon, & Novoplansky, 2006; Fukano & Yamawo, 2015; Gruntman & Novoplansky, 2004). In this case, epigenetic differences between severed individuals of the same genotype, rather than genetic differences, affected self/non‐self‐discrimination. These types of discrimination are not mutually exclusive, and the relative contribution of genetic‐ and epigenetic‐based discrimination may differ among species and situations (Gruntman & Novoplansky, 2004). In *H. tuberosus*, both types of discrimination may be involved. SG pairs shared not only genotype but also physiological environment until seed tubers were cut. The possibility that epigenetic changes in the seed tuber affect self/non‐self‐discrimination can be examined by comparing discrimination between different seed tubers derived from the same maternal plants.

The spatial structure of seedling genotypes may affect the quantity of both asexual (e.g., tubers) and sexual reproduction. Westley (1993) showed that experimental removal of inflorescence buds in *H. tuberosus* increased the size, number, and total biomass of tubers produced per plant. The results suggested a possible trade‐off in resource allocation between sexual and asexual reproduction. We did not quantify flowers and seeds because of the difficulty of their counting in each individual in the field. Plants in self pairs and the AGG group might produce fewer inflorescences than those in non‐self pairs and the ALT group, respectively (Westley, 1993). It would be interesting to investigate the plastic response to neighboring plants not only in terms of altered allocation to competitive and reproductive organs, but also to sexual and asexual reproduction.

Many studies have shown the benefits of intercropping; average yield is often higher in mixtures of multiple species, varieties, or genotypes than the yield in non‐mixed groups (Brooker et al., 2015; Litrico & Violle, 2015). This is the case if the resource competition among the neighboring plants of mixed groups is smaller than non‐mixed groups (resource use complementary). On the other hand, if the resource competition among the neighboring of mixed groups is the same as or greater than among non‐mixed groups, and/or if the plants are able to increase their allocation to competitive traits depending on the genetic relatedness of neighboring plants, the mixed groups will reduce the resource use efficiency and production, as suggested by the results of the present study. Thus, the benefits of spatially heterogenous (i.e., intercropping) or homogenous planting (i.e., genotype‐aggregated planting) will depend on the degree of competition for resources and the response of plants based on kin or self‐discrimination among the neighboring plants.

In many parts of the world, people cultivate open‐pollinated and landrace crops (Almekinders, Louwaars, & Debruuijn, 1994), which maintains genetic diversity within cultivars and populations. In such agricultural systems, farmers might be able to increase yield by arranging the spatial structure of seedling genotypes. Because this method can increase yields without the need for additional fertilizers or pesticides and without expansion of agricultural area, it may contribute to sustainable farming practices. However, genotype‐aggregated planting might decrease the yield in the field under pathogen infection or abiotic stresses. If the susceptibility (including mortality) of each genotype to these stresses varies, spatial distribution of the dead plants should differ between AGG and ALT planting. The space freed by dead plants may affect the growth and reproduction of the neighboring survivors and therefore field‐level yield. Further studies are needed to determine the effectiveness and limitations of genotype‐aggregated planting methods in various crop systems under field conditions.

## DATA ARCHIVING STATEMENT

Data for this study are available from the Dryad Digital Repository: https://doi.org/10.5061/dryad.r2t034g
