# Supplementary figures and images for: Genotype‐aggregated planting improves yield in Jerusalem artichoke (Helianthus tuberosus) due to self/non‐self‐discrimination
Source: Evol Appl. 2018 Nov 29;12(3):508–18. doi: 10.1111/eva.12735 (PMC6383731; doi:10.1111/eva.12735)

(a)

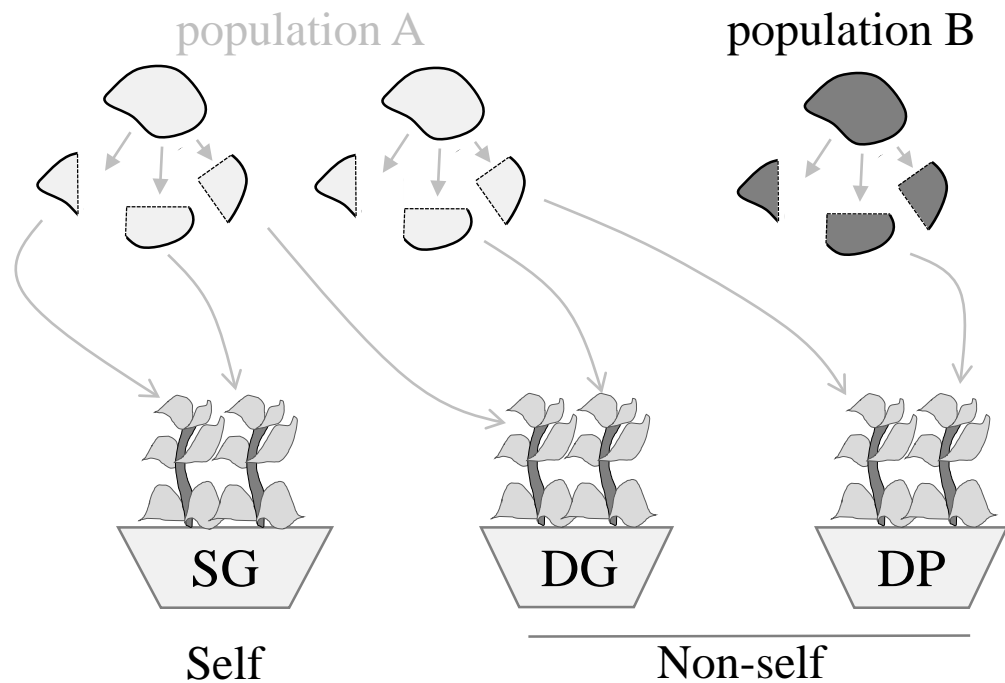

(b)

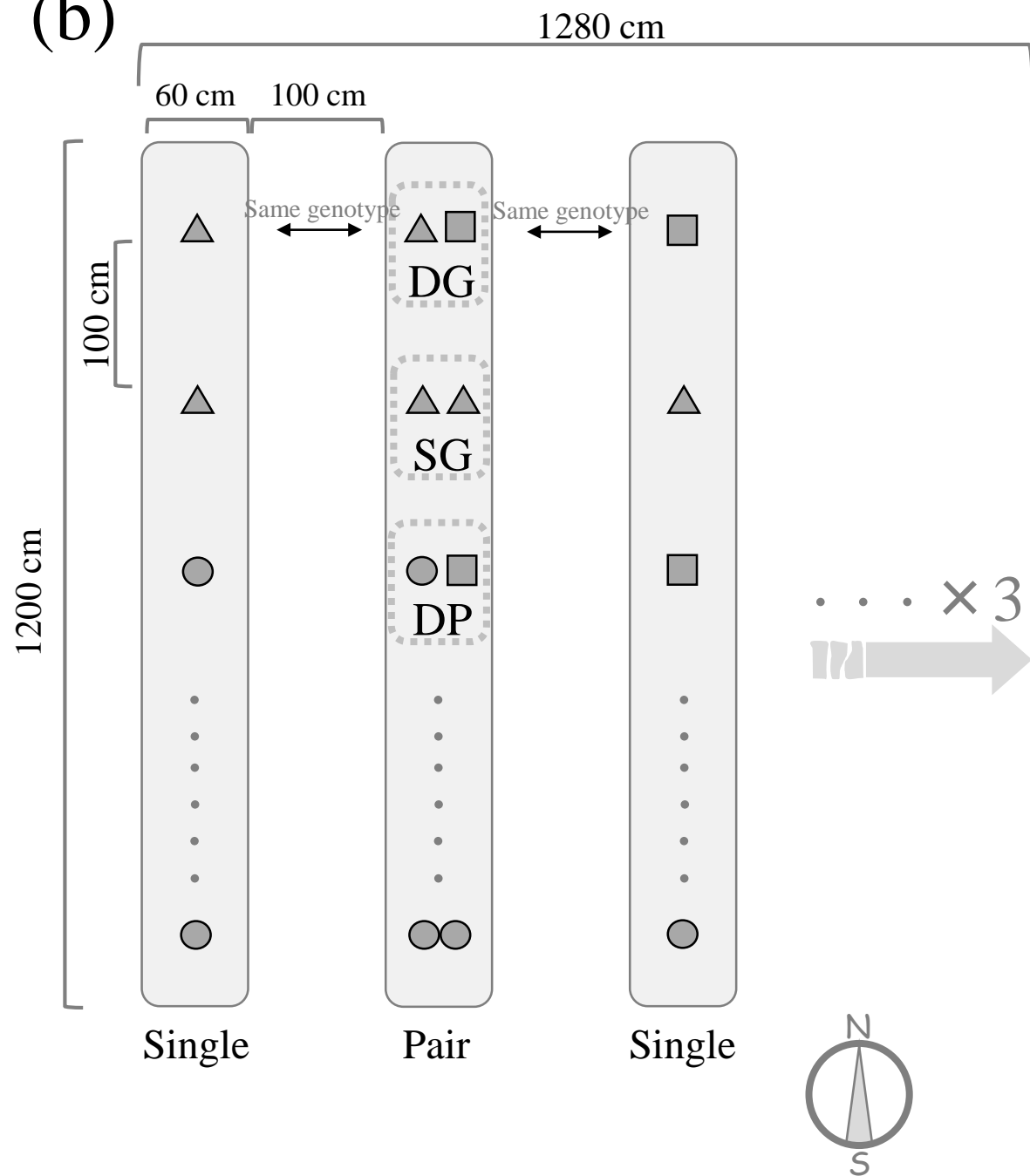

(c)

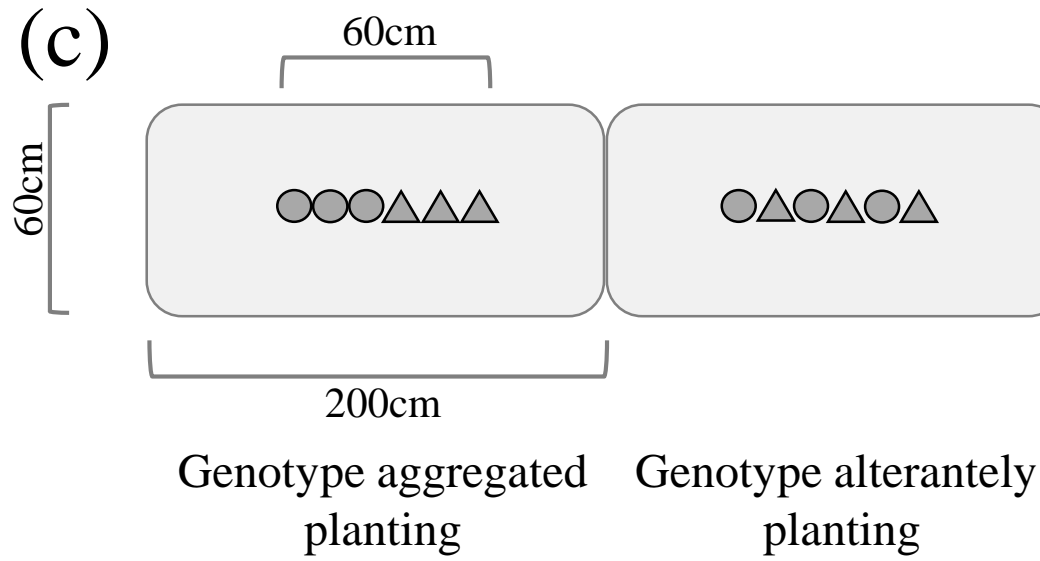

Supplement: Supplementary file 1 [file EVA-12-508-s001.pdf]

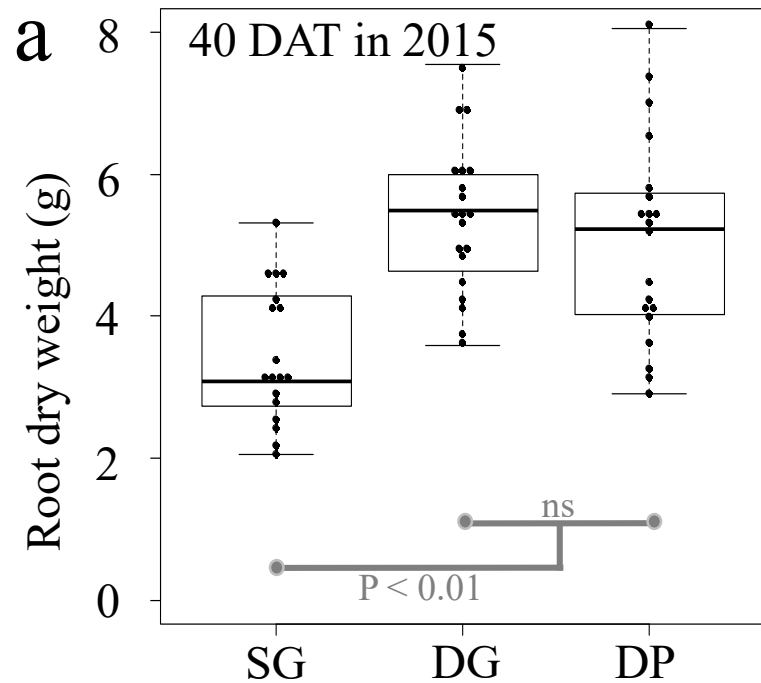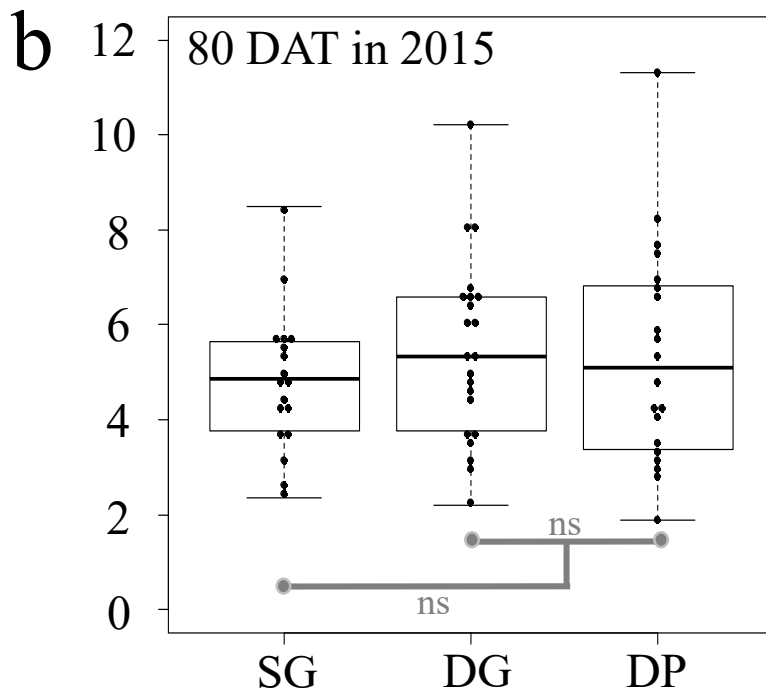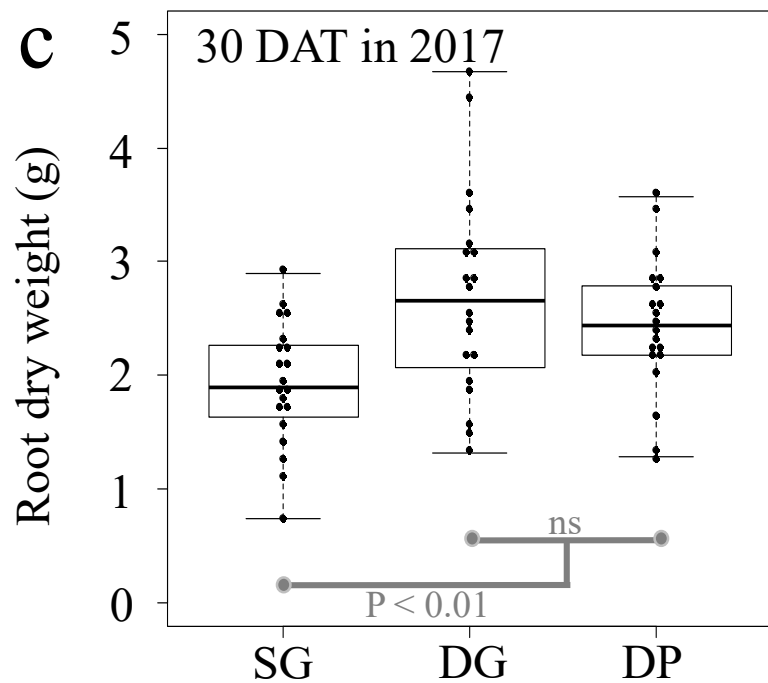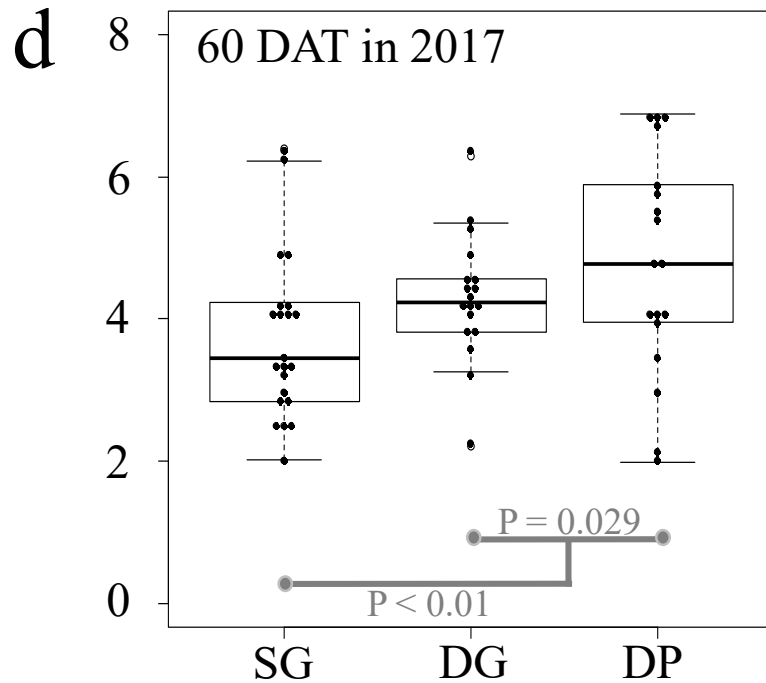

Supplement: Supplementary file 2 [file EVA-12-508-s002.pdf]

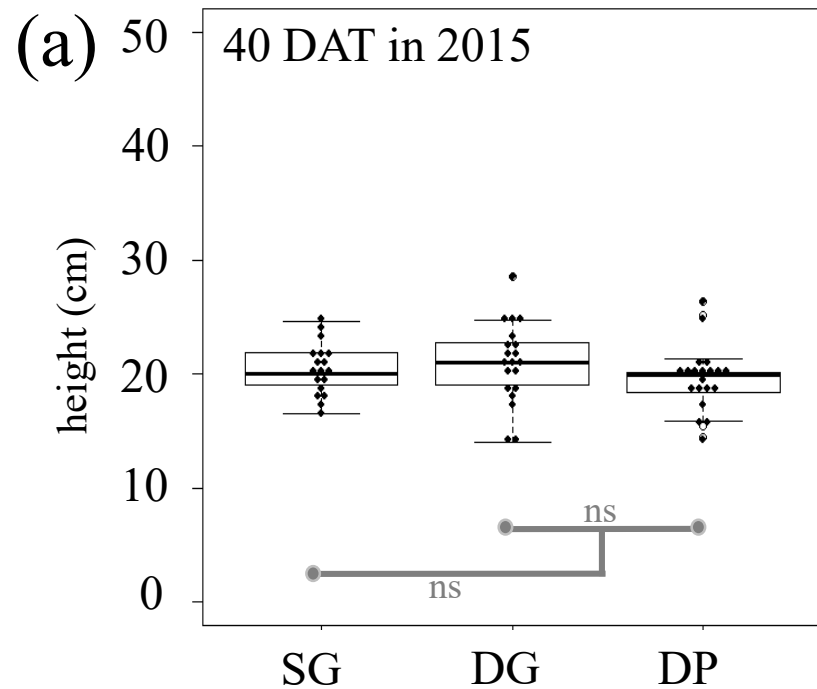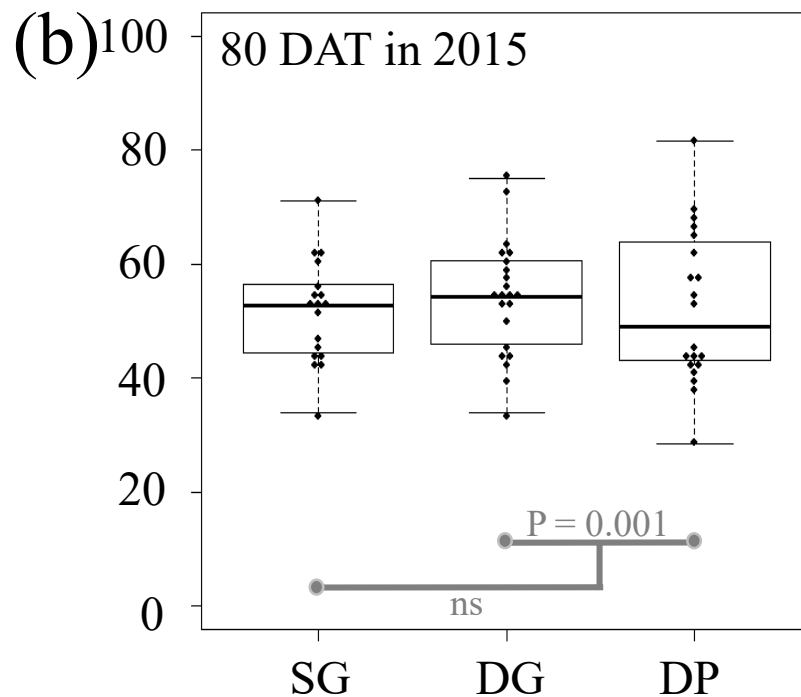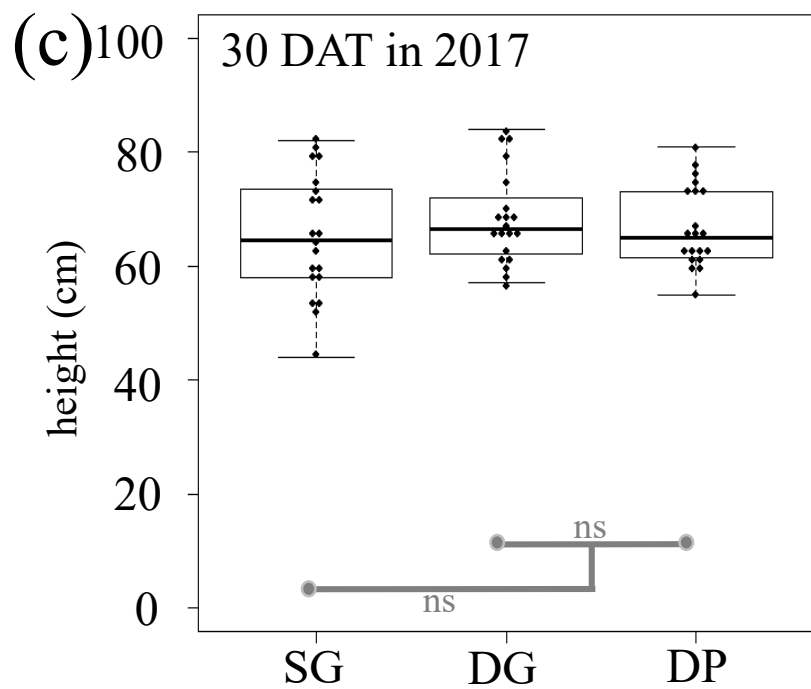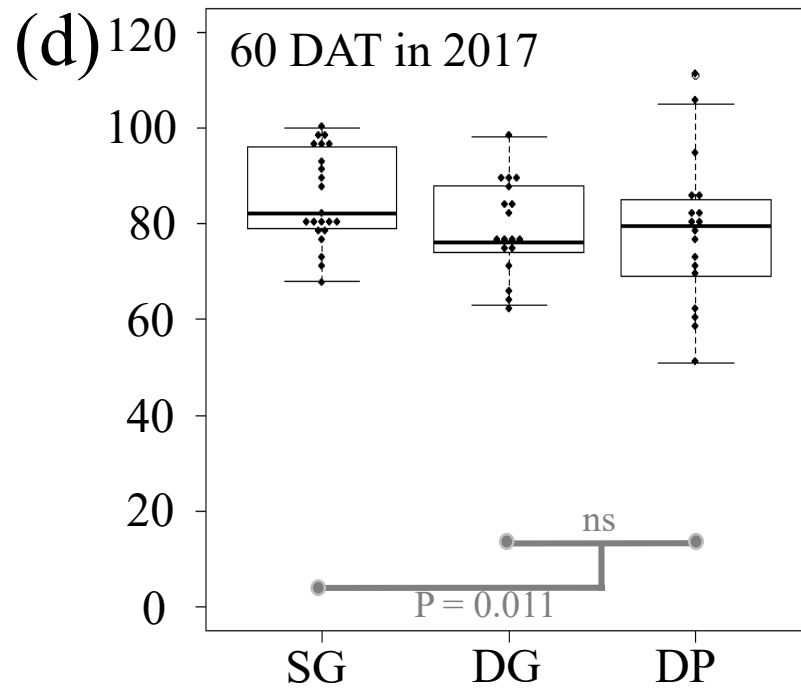

Supplement: Supplementary file 3 [file EVA-12-508-s003.pdf]

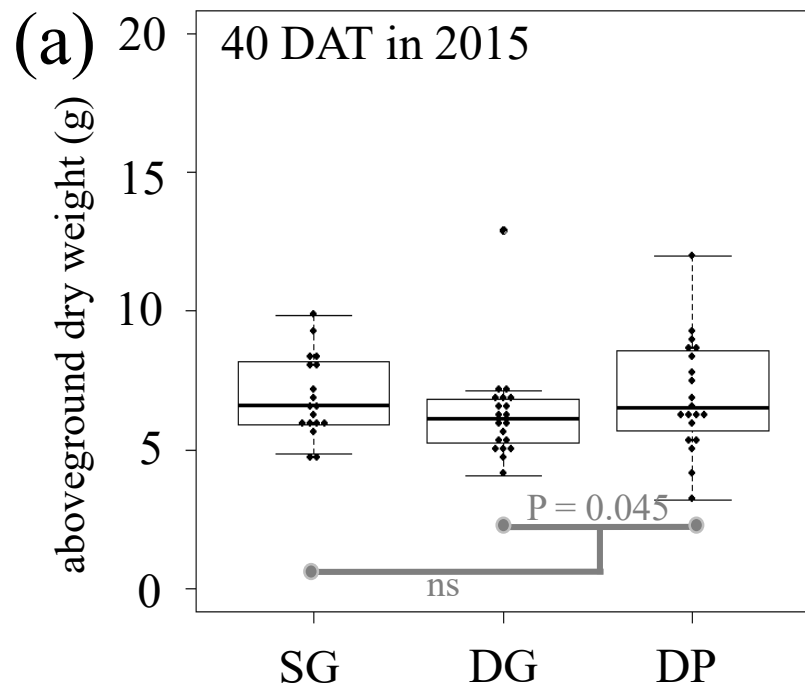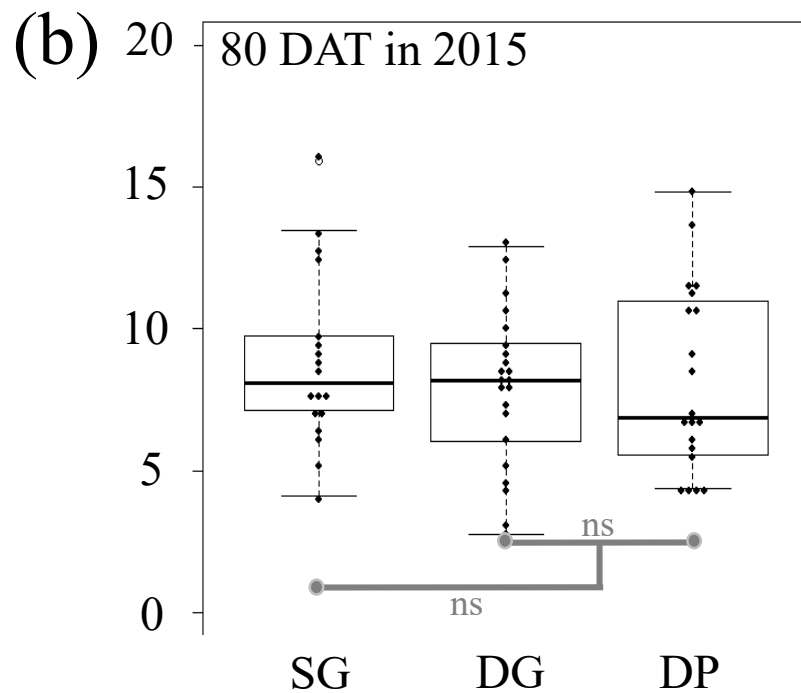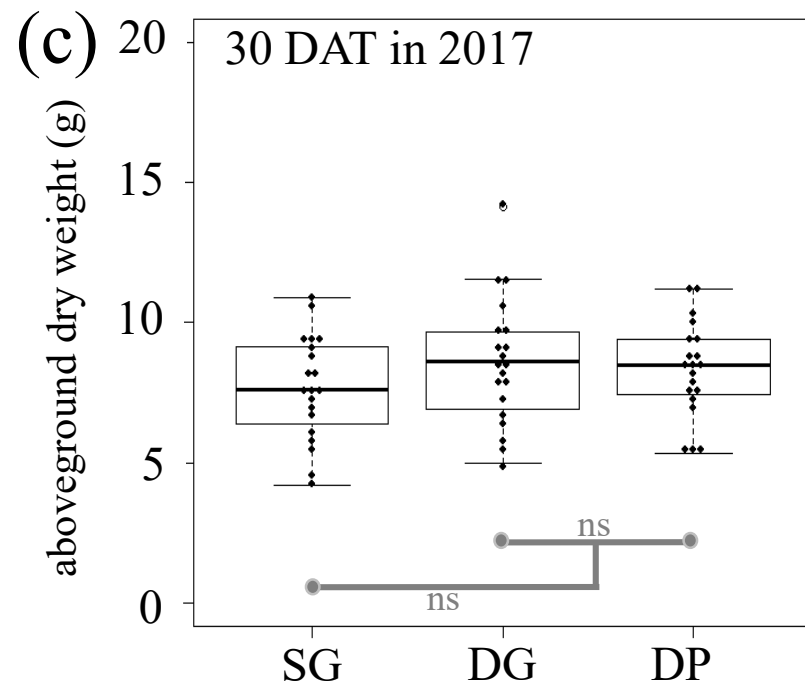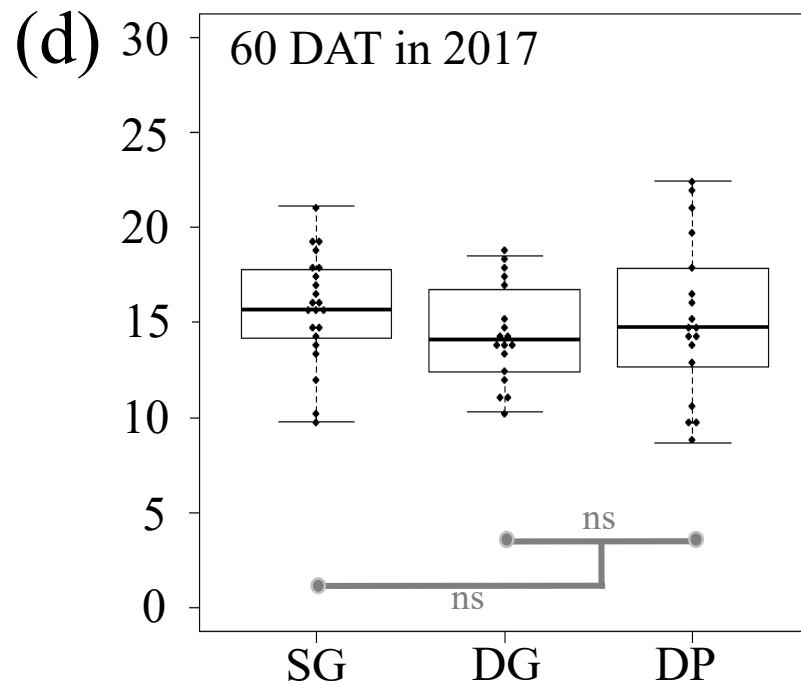

Supplement: Supplementary file 4 [file EVA-12-508-s004.pdf]

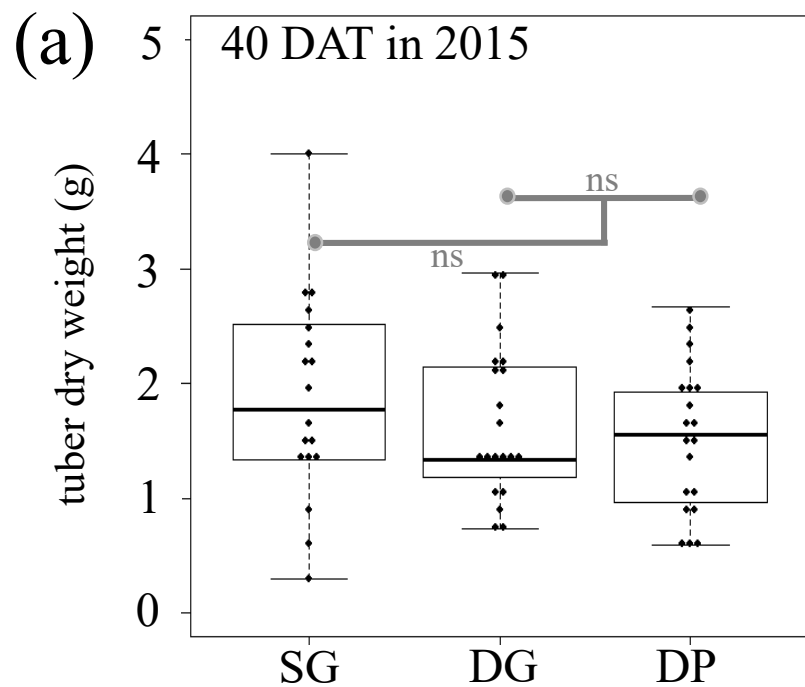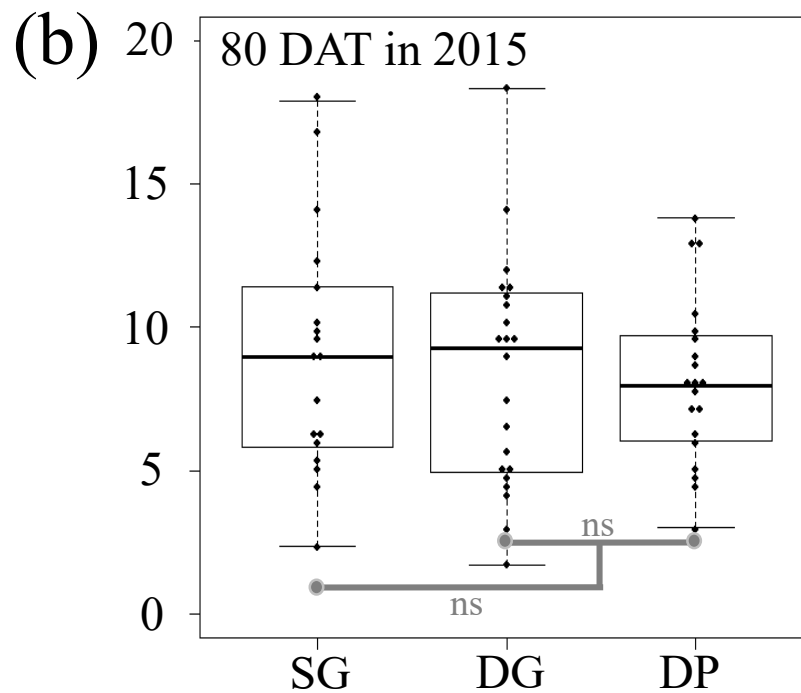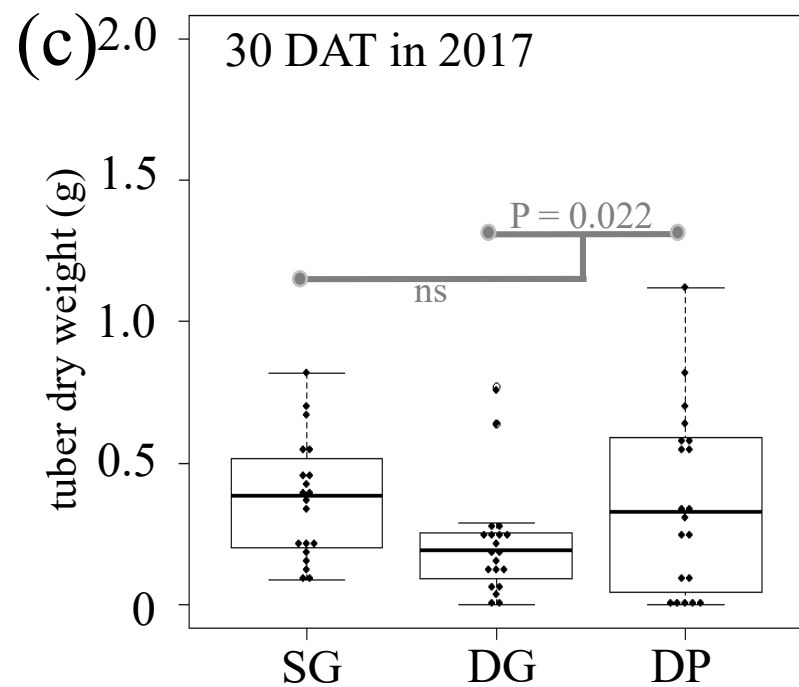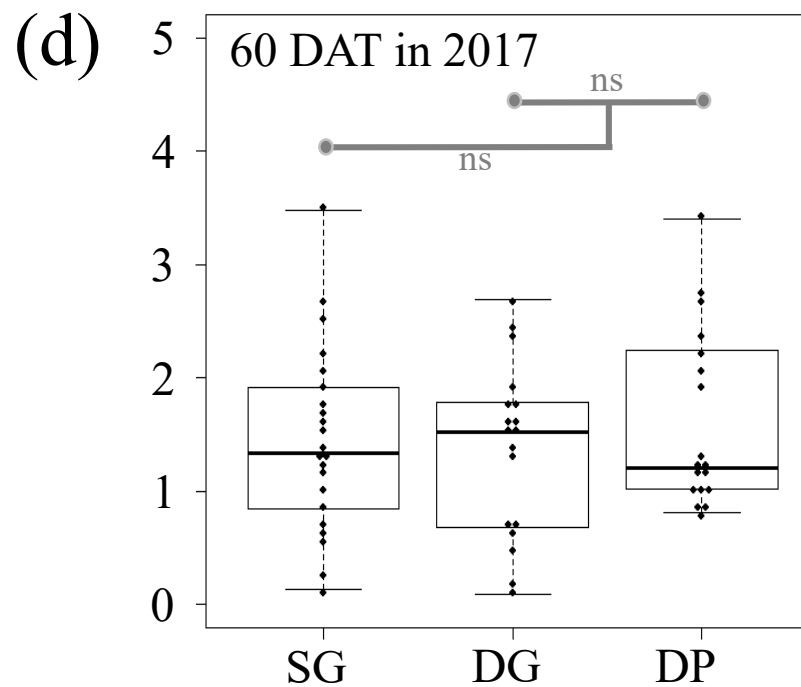

Supplement: Supplementary file 5 [file EVA-12-508-s005.pdf]

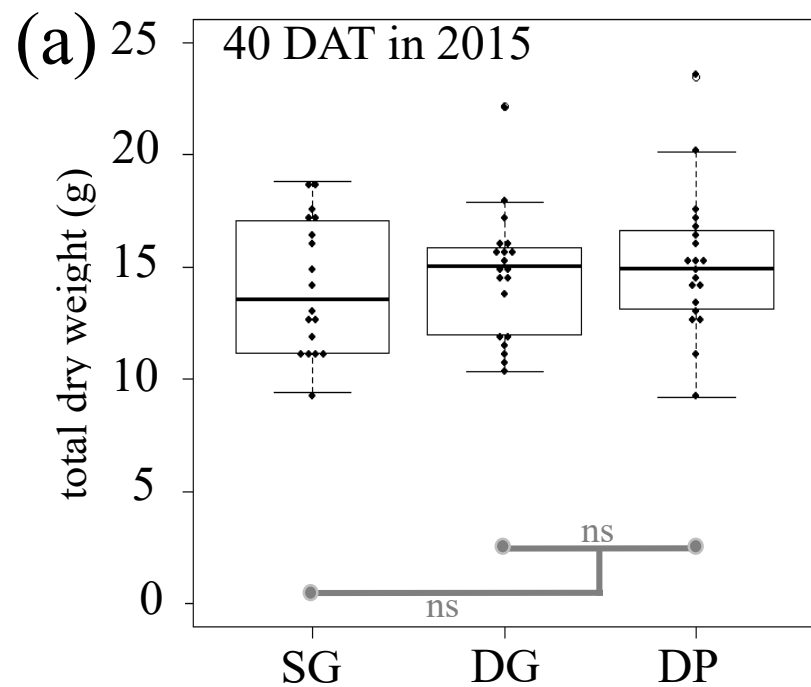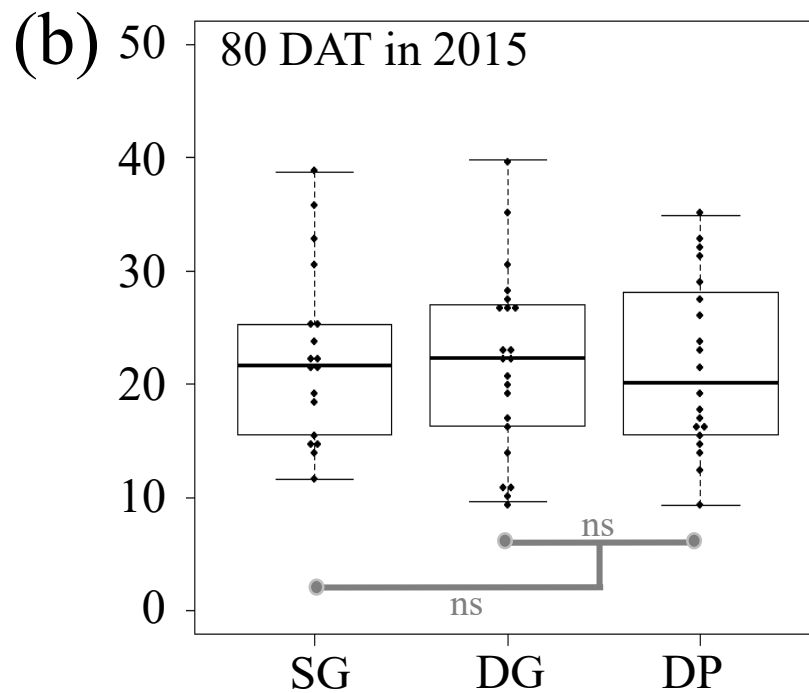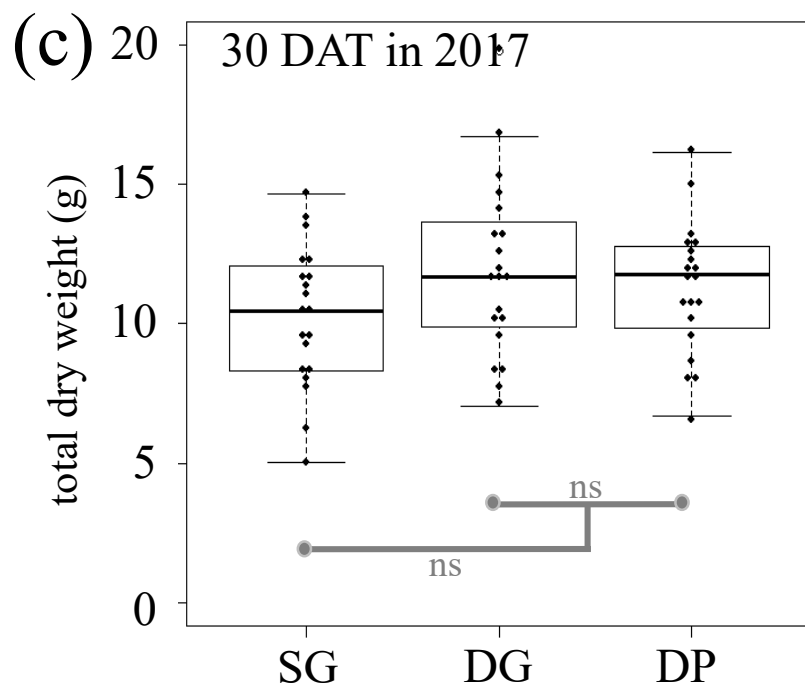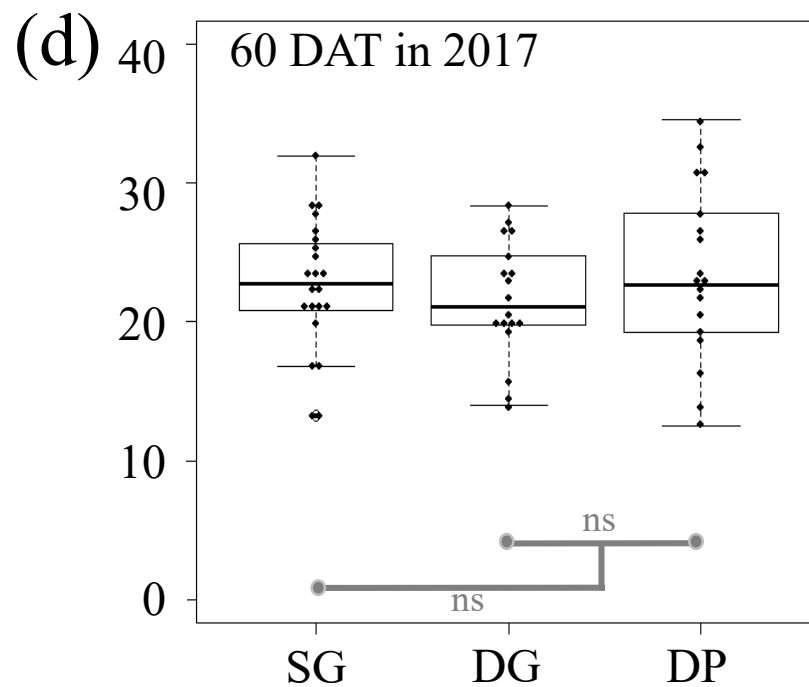

Supplement: Supplementary file 6 [file EVA-12-508-s006.pdf]

(a) 40 DAT in 2015

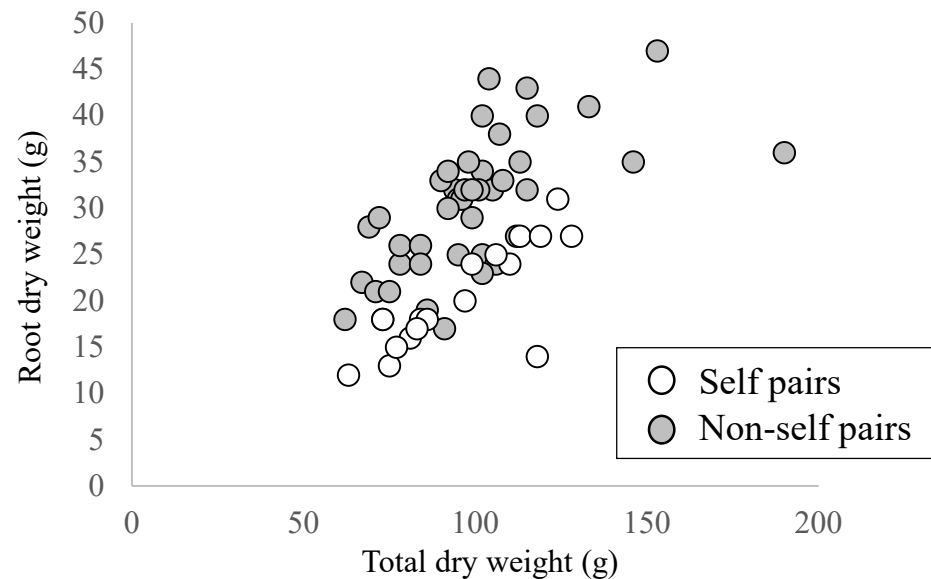

(b) 80 DAT in 2015

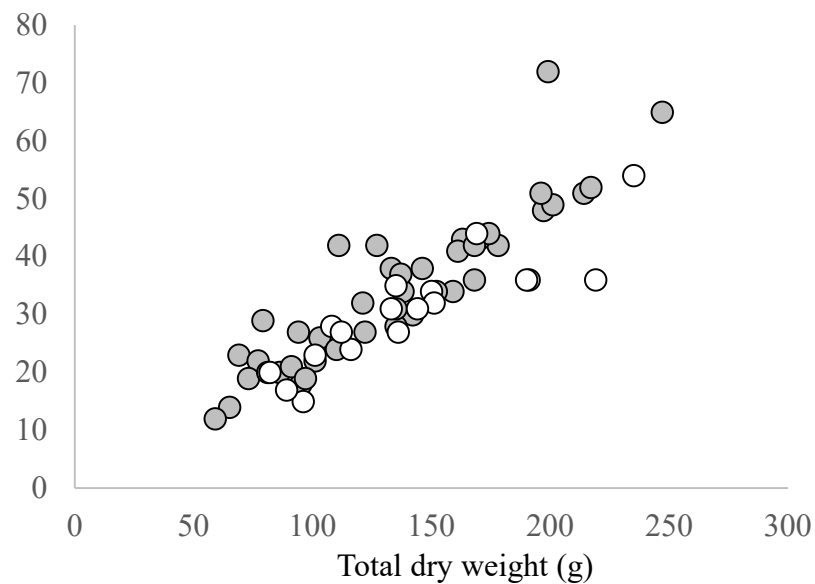

(c) 30 DAT in 2017

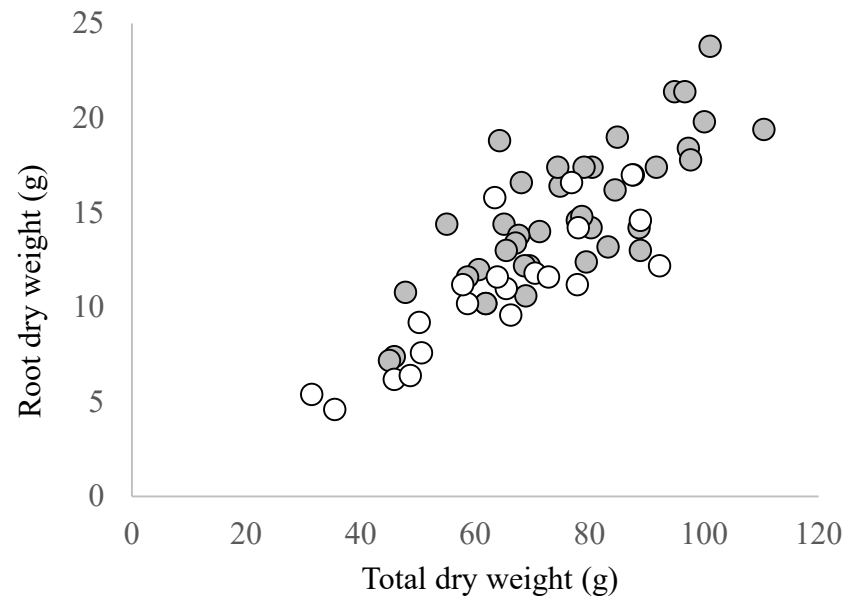

(d) 60 DAT in 2017

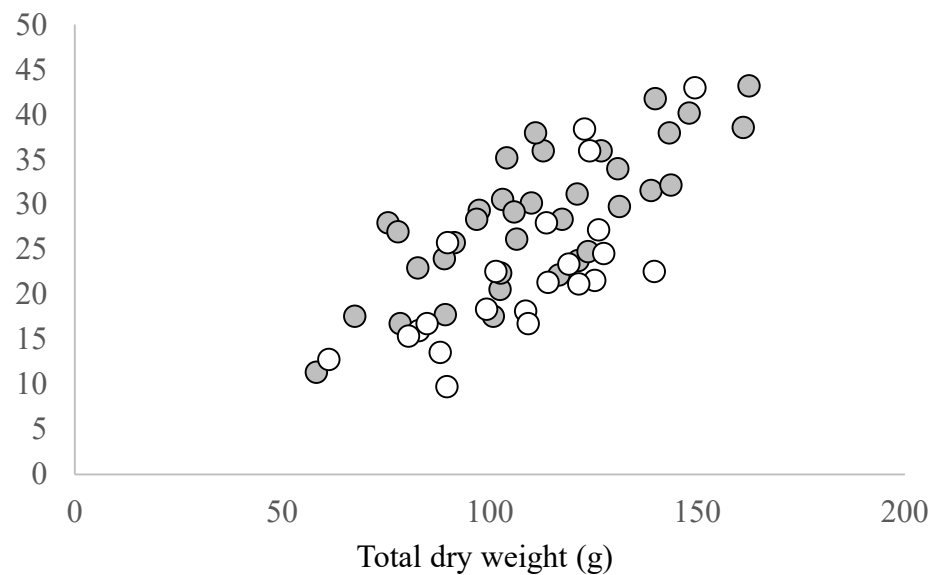

Supplement: Supplementary file 7 [file EVA-12-508-s007.pdf]

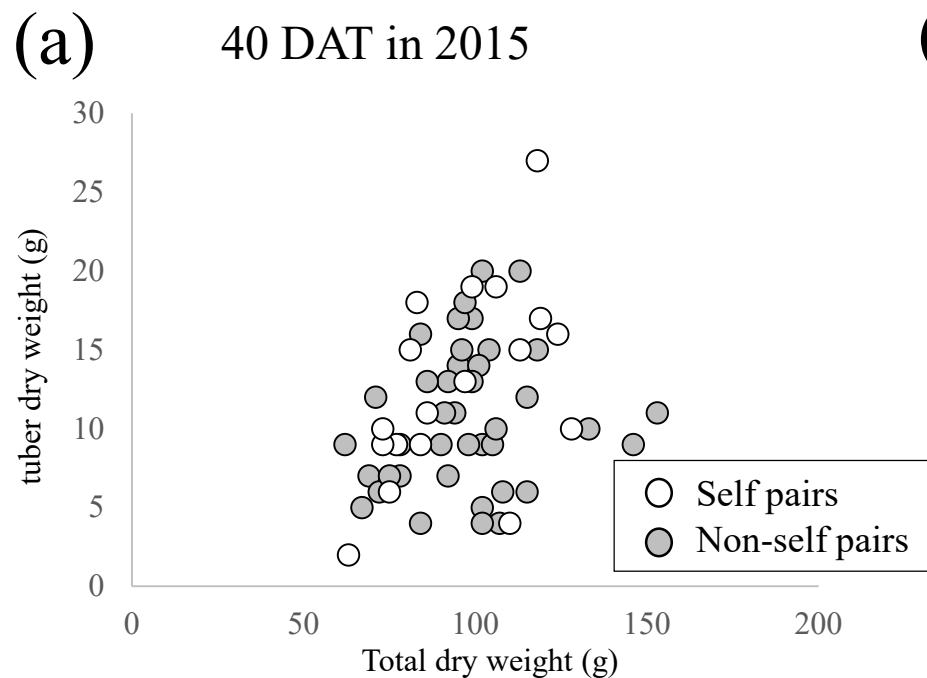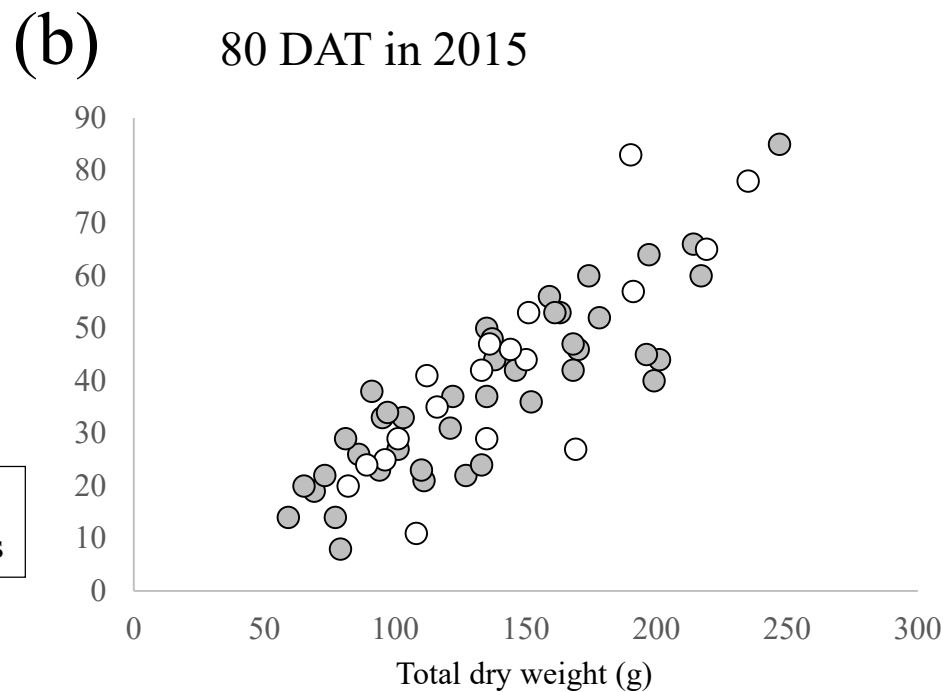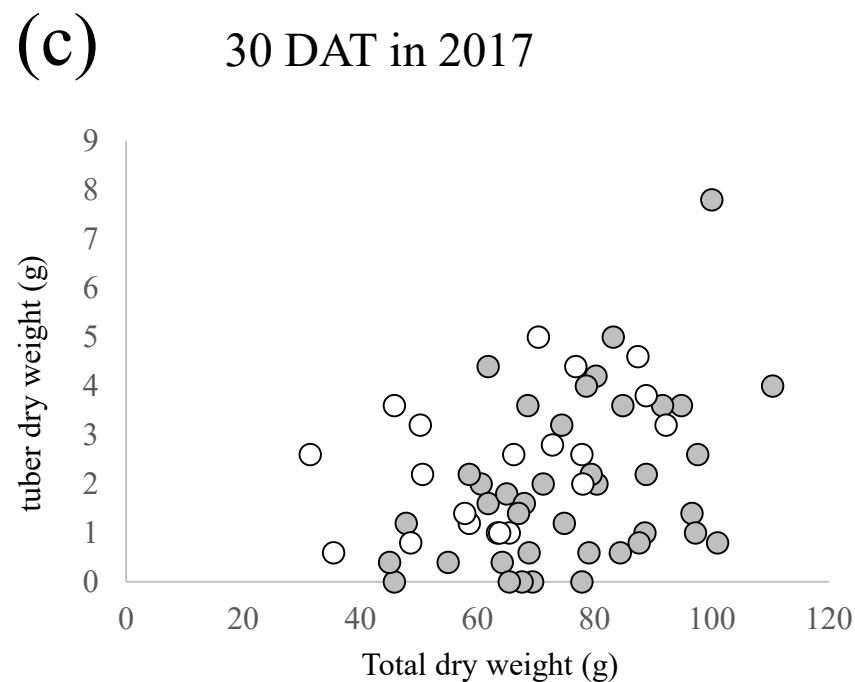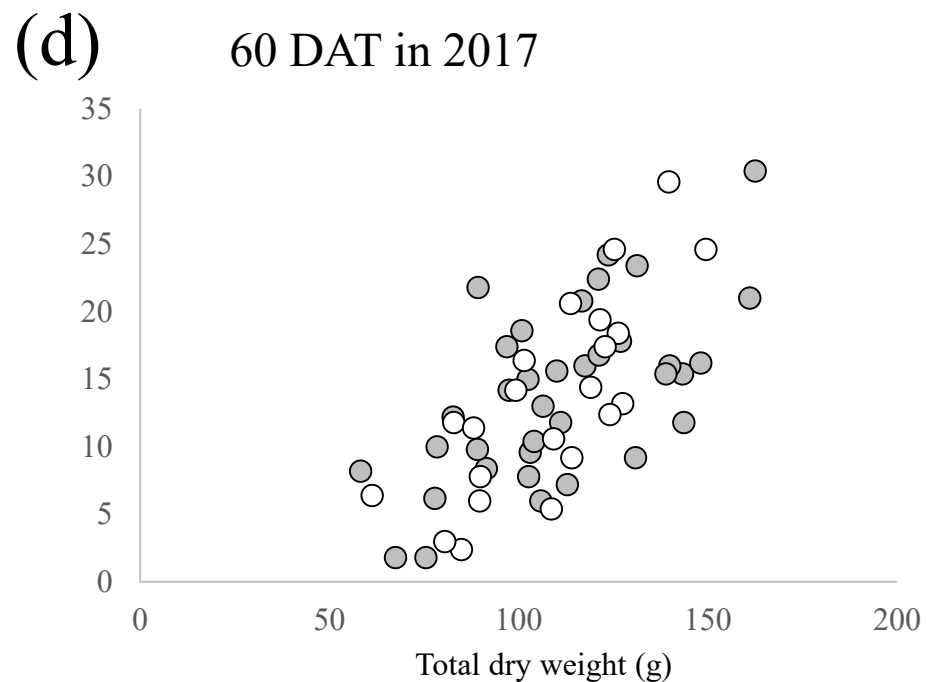

Supplement: Supplementary file 8 [file EVA-12-508-s008.pdf]

(a) 40 DAT in 2015

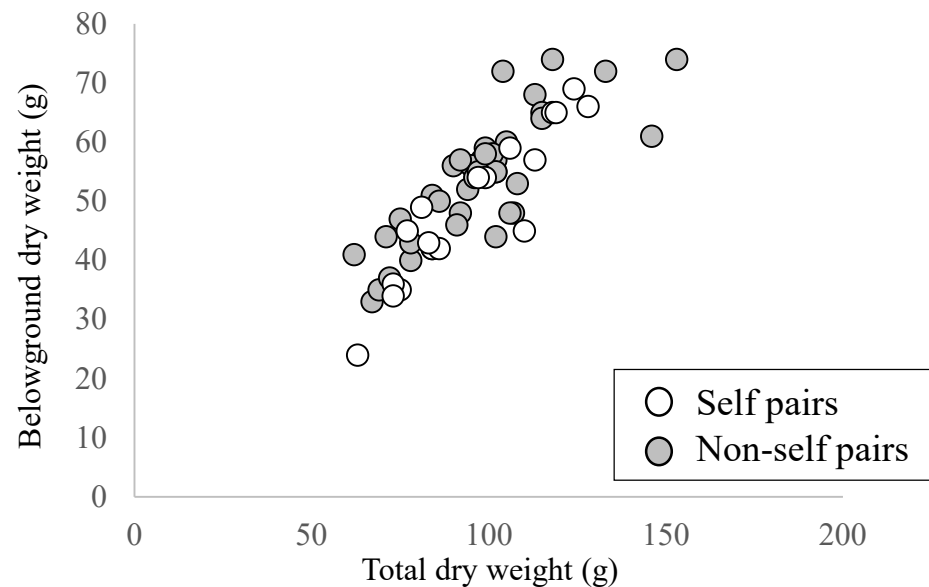

(b) 80 DAT in 2015

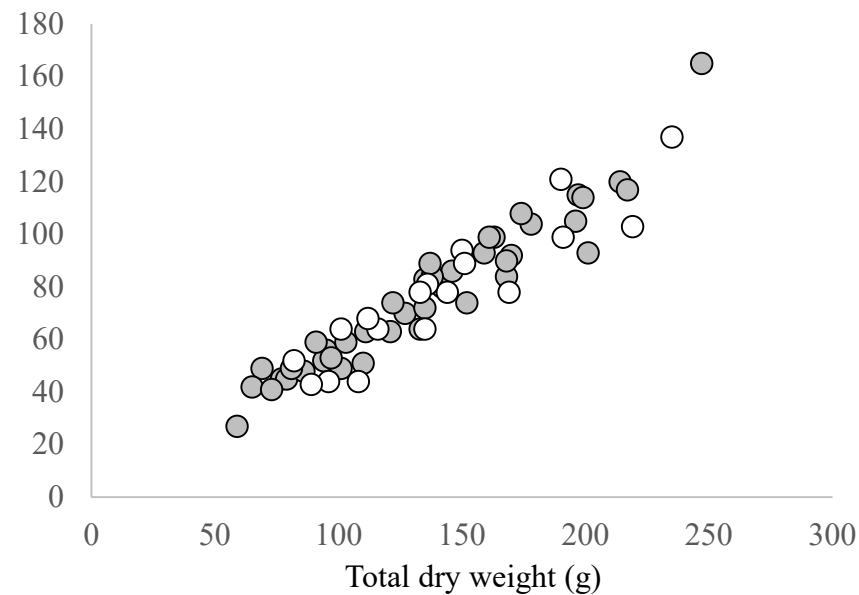

(c) 30 DAT in 2017

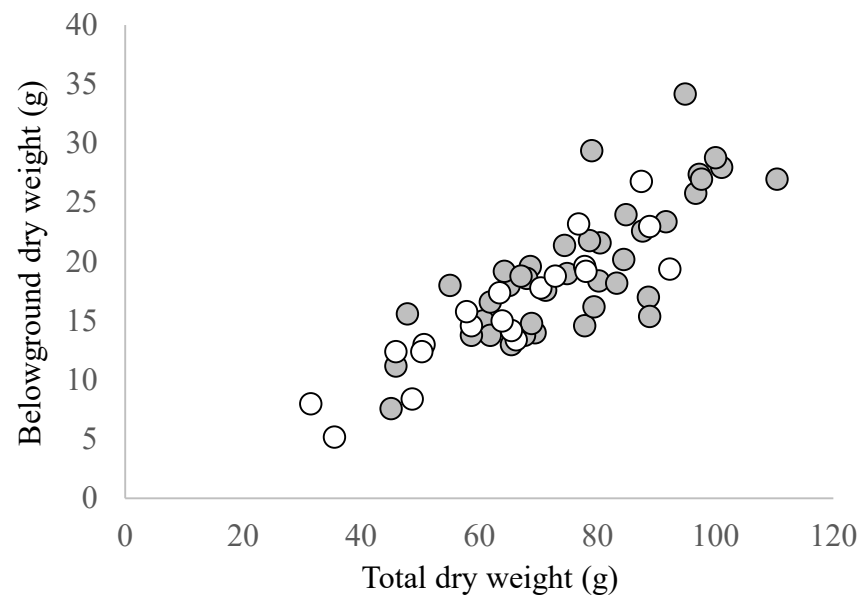

(d) 60 DAT in 2017

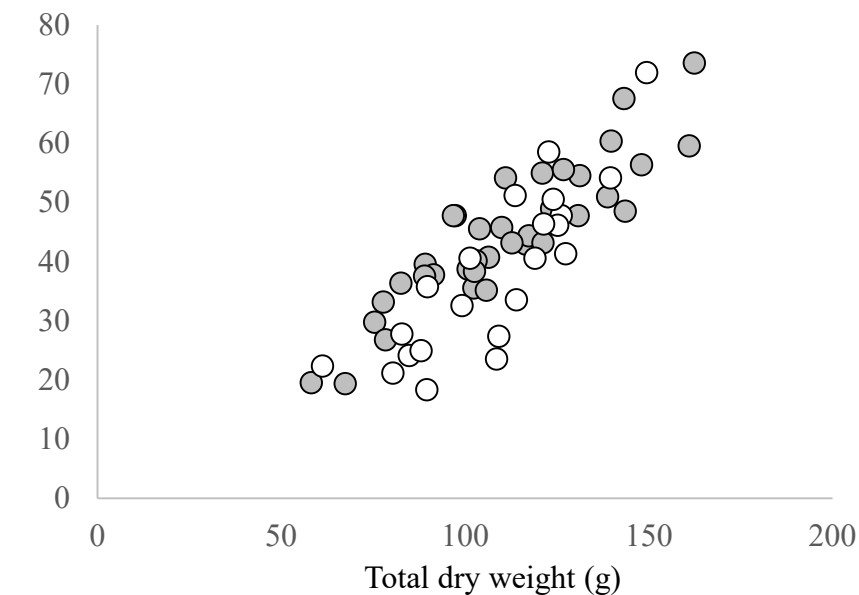

Supplement: Supplementary file 9 [file EVA-12-508-s009.pdf]
